# The College Commencements

**Published:** 1892-06

**Authors:** 


BUFFALO MEDICAL AND SURGICAL JOURNAL
A MONTHLY REVIEW OF MEDICINE AND SURGERY.

*EDITORS:*
THOMAS LOTHROP, M. D. - - W. WARREN POTTER, M. D.
All communications, whether of a literary or business character, should be addressed to the managing editor: 284 FRANKLIN STREET, BUFFALO, N. Y.

VOL. XXXI. JUNE, 1892. No. 11.

### THE COLLEGE COMMENCEMENTS.

THE NIAGARA UNIVERSITY MEDICAL COLLEGE held its graduating exercises at the Star Theatre, on Monday evening, May 2d, where a large audience was assembled to witness the proceedings. The Rt. Rev. Bishop Ryan, Chancellor of the University, presided, and President Cronyn of the Faculty introduced the exercises in a few brief remarks. The degree of Doctor of Medicine was conferred, according to the English hooded rite, upon the following-named candidates: Frederick A. Hayes, Buffalo; Alois Jokl, Buffalo; James Edward Culbert, Scio; James Joseph McAvoy, Waterbury, Conn.; James Joseph Kane, Buffalo; Charles Strong Dickenson, Johnson's Creek; Joseph Henry Dunigan, Greenwood; James Martin Sweeney, New Orleans, La. The Rev. Wm. Burnett Wright delivered the address to the graduating class, which was received with enthusiasm. Then followed the banquet at The Genesee, which was served with much elaboration, and was an occasion of great interest. Dr. J. J. Twohey acted as toastmaster, who announced in their order the following speakers: Medical Department of Niagara University, Professor Thomas Lothrop; The Clergy, Rev. Andrew E. Purdy; The Legal Profession, D. J. Kenefick; Niagara University, Literary Department, Professor Grace; The Press, by Leslie Thom; Valedictory Address by Dr. J. J. McAvoy, of the graduate class.

The Alumni Association held its meeting at the college building during the day, at which time papers were read as follows: A Conservative View of the Treatment of Appendicitis, Illustrative Cases, by N. G. Richmond, M. D., Fredonia, N. Y.; Gallstones without Icterus, by C. B. LeVan, M. D., Buffalo, N. Y.; Influenza, with Microscopic Demonstrations of the Recently Discovered Bacillus, F. J. Thornbury, M. D., Buffalo, N. Y.

This brief synopsis does but imperfect justice to the ceremonies of this interesting occasion, which concluded one of the most satisfactory years in the history of this college.

---

The Medical College of Buffalo University held its forty-fifth annual commencement on Tuesday evening, May 3d, at Music Hall. The degree of Doctor of Medicine was conferred upon the following-named graduates:

Thomas P. C. Barnard, Walter Steele Barnes, Edward B. Belknap, William G. Bissell, Charles E. Boult, Clara E. Bowen, Arthur R. Bradbury, A. M., Warner C. Bush, N. Victoria Chappell, Alexander L. Ladd, Milton G. Lape, Ada C. Latham, Emma C. LeFevre, Barton T. McDowell, Albert H. Macbeth, James F. Macpherson, Marion E. Martin, Henry H. Mayne, William J. Chisholm, Ferdinand G. Moehlau, Amanda M. Congdon, Nellie Edmonds Murray, Mortimer S. Coon, Samuel R. Peacock, Andre M. Cowles, M. D., Howard A. Pease, Arland Lewis Darling, John C. Preston, Archie I. Drake, Benjamin W. Readshaw, Henry A. Eastman, Reuben A. Reeves, William Follett, Jr., Hudson M. Roberts, William C. Fritz, William Z. Roberts, Edward L. Frost, Lamont H. Ross, Maud Josephine Frye, Lister Gibbons, Morris S. Hadsall, George J. Hearne, Gustavus A. Hitzell, George S. Hobbie, Harry Corwin Jones, Wirt W. Jones, C. Oakley Sayres, Owen M. Shreve, Ph. B., Edward E. Stanbro, Ph. G., George B. Stocker, Andrew J. Volker, Charles F. Wixom, John V. Woodruff and Samuel W. Worrell.

The degree of Doctor of Pharmacy was conferred upon the following-named candidates:

Emery Jay Bargar, George Orrin Baxter, James Rollin Brown, Udell Sylvester Braman, Charles Wilton Chase, William Edward Delehant, Burt F. Disbrow, Margaret Elizabeth Fisher, Earl Look Hess, Max Lothar Kaesnter, William Arnold Kendall, James Bryden Mason, Adelbert Coon Miller, Rosa Belle Mosher, William Burt Reed, Edward James Rogers, Frank Rowley, Jr., John Timothy Sanford, Ernest Everett Vahey, Ernest Brice Walker, and Benjamin Howard Westgate.

The address to the graduating classes was delivered by Dr. George M. Gould, of Philadelphia, editor of the *Medical News*, and the address to the Alumni was made by Professor James Law, of Cornell University. After the exercises at Music Hall were concluded, the annual banquet of the Alumni was served at The Genesee. Dr. M. B. Folwell presided, and toasts were responded to by the Hon. E. C. Sprague, the Rev. Wm. S. Hubbell, Dr. Charles S. Butler, the Rev. Frank S. Fitch, Mr. George E. Vincent, and Dr. J. V. Woodruff.

The annual meeting of the Alumni was held at the college building during the day, and a large number of the graduates attended. Papers were read on Parasites by Dr. H. D. Walker; Clinical Instruction in Obstetrics, by Dr. P. W. Van Peyma; Hysteria in Children, by Dr. J. W. Putnam; and One Not Well-known Cause of Conjunctivitis, by Dr. Julius Pohlman.

At the meeting of the Council, held in the afternoon, arrangements were made for the organization of a department of dentistry, which will be ready for the reception of students in the Autumn. The instructors chosen are Drs. William C. Barrett, Alfred P. Southwick, Herbert A. Birdsall, and Frank E. Howard. This proved one of the most satisfactory gatherings of the Alumni, and one of the most interesting commencement occasions in the history of the college.

